# Biomantling and Bioturbation by Colonies of the Florida Harvester Ant, *Pogonomyrmex badius*

**DOI:** 10.1371/journal.pone.0120407

**Published:** 2015-03-20

**Authors:** Walter R. Tschinkel

**Affiliations:** Dept. of Biological Science, Florida State University, Tallahassee, Florida, United States of America; Arizona State University, UNITED STATES

## Abstract

In much of the world, soil-nesting ants are among the leading agents of biomantling and bioturbation, depositing excavated soil on the surface or in underground chambers. Colonies of the Florida harvester ant, *Pogonomyrmex badius* excavate a new nest once a year on average, depositing 0.1 to 12 L (3 L average) of soil on the surface. Repeated surveys of a population of about 400 colonies yielded the frequency of moves (approximately once per year), the distance moved (mean 4 m), and the direction moved (random). The area of the soil disc correlated well with the volume and maximum depth of the nest, as determined by excavation and mapping of chambers. The population-wide frequency distribution of disc areas thus yielded the frequency distribution of nest volumes and maximum depths. For each surveyed colony, the volume of soil excavated from six specified depth ranges and deposited on the surface was estimated. These parameters were used in a simulation to estimate the amount of soil mantled over time by the observed population of *P*. *badius* colonies. Spread evenly, *P*. *badius* mantling would create a soil layer averaging 0.43 cm thick in a millennium, with 10–15% of the soil deriving from depths greater than 1 m. Biomantling by *P*. *badius* is discussed in the context of the ant community of which it is a part, and in relation to literature reports of ant biomantling.

## Introduction

Exposed to the elements at the surface of the earth, parent rock is weathered into the components of soil, and these components can accumulate in place or be transported and deposited by water and wind into stratified layers organized by their history and age. Historically, soil scientists have emphasized the chemical, physical and geological processes involved in soils. For example, water percolating through soils influences the weathering of parent rock, and transports soluble minerals to deeper layers where they can be deposited in various forms or lost by transport in ground water. Water and wind create soil erosion, processes that are strongly affected by slope and soil texture. Relatively recently, emphasis has increasingly shifted to biological processes. Soils around the world are home to a large fauna and flora that change the character of the soil they live in. Microbes and plants contribute to continued weathering, extraction, creation of pores and displacement of soluble components. Animals that burrow in soils move masses of buried soil to the surface (biomantling) or to other levels below the surface (bioturbation), greatly complicating the stratification of the soil that is their home, and affecting the physical and chemical processes that modify soils and their stratification. Bioturbation and biomantling, to various degrees, interrupt or enhance these physical and chemical processes because they move mineral nutrients from deeper layers to shallower layers or the surface, often benefiting the growth of plants. The list of animals that live in and stir the soil is long indeed, ranging from worms who eat their way through soil, to arthropods in their endless variety to aardvarks and their palatial burrows.

The study of bioturbation began with Darwin's [1] observations that objects on the soil surface slowly became buried as the result of the activity of earthworms [2]. A review of the early post-Darwin literature can be found in Johnson and Johnson [3]. During the following century and a half, the roles of animals and plants in modifying, mixing and layering soils has become increasingly recognized and quantified [4]. Mounding of soil by burrowing vertebrates affects not only mixing and turnover, but also subsequent down-slope movement of soils [5]. Invertebrates such as ants affect the heterogeneity of soil composition and texture by preferential movement of different size grains [6], followed by further sorting by rainwash [7–9]. The macropores created by invertebrate burrowers affect water infiltration [9, 10–11] as well as runoff [6] and modify the soil "fabric" and texture [12, 13]. Moreover, trace fossils of Miocene age emphasize that burrowing animals have been modifying soils for a very long time [14].

Bioturbation is of particular concern to archeologists and earth scientists because it mixes and inverts soil layers, complicating artifact stratification and radiocarbon dating [15, 16]. Biomantling and turbulent bioturbation result in the burial of objects on the soil surface, such as artifacts, tiles [17] or cracked pavement [3]. When animals move sand from one soil layer to another without exposing the grains to light, they affect the dates derived from optically stimulated luminescence [18, 19], creating complications and challenges for archeologists.

Surely the most consistent soil-turners, based on their sheer abundance and importance in terrestrial ecosystems are the ants. A large proportion of the 14,000 described species excavate nests in the soil, mostly disposing of the waste soil on the surface, but also remodeling their underground abodes [18, 20, 21]. Comparison among bioturbating animals found the mounding rates of ants to be comparable or higher than termites, earthworms and burrowing vertebrates [5, 22–23]. Frequent nest relocation by ants magnifies their effect [11, 22, 24]. The magnitude of ant action is sometimes as obvious as the colossal mound nests of the European *Formica* species, and the earthen mounds of fire ants [25], Allegheny mound builders or the attine leafcutter ants in the genera *Atta* and *Acromyrmex* [3, 26]. Several reports have estimated the amount of mantling by species of ants or entire ant communities per m^2^ /yr and/or per nest mound/yr [3, 6, 27]. Eldridge and Pickard [11] estimated that the entire soil profile would be turned over in 200 years by an Australian *Aphaenogaster* species, explaining why these soils had little horizon development. But even inconspicuous species with small colonies can turn remarkable amounts of soil because of the sheer abundance of their nests, as for example *Trachymyrmex septentrionalis* [28] and Australian *Aphaenogaster* "funnel ant" spp. [22]. Baxter and Hole[29] found 1531 mounds of the ant *Formica cinerea montana* per ha, covering about 1.7% of the surface area, and suggested that the ants bring up a substantial amount of soil from lower soil horizons. In a 25 yr study, Dorn [30] showed that ants enhance the weathering of basaltic calcium-magnesium silicates into carbonates by 50 to 300 fold over controls, speculating that ants may play a role in atmospheric carbon dioxide sequestration.

Reliable estimates of the rate of biomantling and bioturbation by ants require several pieces of information that rarely occur together. One must know the volume of the excavated nests (many reports use mound volume as a proxy) and how that volume is distributed with depth [31]; one must know the density of nests in the habitat, and their size-frequency distribution; one must know the frequency with which ants excavate new nests and the rate at which they do so. Finally, one must know whether all excavated soil is dumped on the ground surface, or whether some proportion is redeposited underground by filling previously excavated chambers or some other form of deposition [18, 28]. All of these conditions are met by the Florida harvester ant, *Pogonomyrmex badius*, a large, charismatic and characteristic ant of the coastal plains and piedmont of the eastern USA from Louisiana to North Carolina [32]. Since 2010, we have resurveyed a dense population of *P*. *badius* several times a year, collecting all the necessary data that, when combined with Tschinkel's [31] description of nest architecture, allowed a detailed data-rich estimate of biomantling and bioturbation by *P*. *badius*. A description of the process and parameters of nest relocation in *P*. *badius* is available in Tschinkel [33].

## Materials and Methods

### Study site

This study was carried out under permit number APA583 and APA56302 from the US Forest Service, Apalachicola National Forest. The study population of Florida harvester ant, *Pogonomyrmex badius*, is located in a 23 ha site (latitude 30.3587, longitude −84.4177) about 16 km southwest of Tallahassee, Florida, USA, within the sandhills portion of the Apalachicola National Forest. The site, Ant Heaven, consists of well-drained sandy soil occupying a slope to a wetland and stream, causing its water table to be depressed (>5 m at the maximum), thereby making it suitable for *P*. *badius* and *Solenopsis geminata*, as well as several drought-resistant species of plants such as *Opuntia* and *Nolina*. The area also supports a population of gopher tortoise (*Gopherus polyphemus*). The forest consists of longleaf pines (*Pinus palustris*) planted ca. 1975, turkey oak (*Quercus laevis*), bluejack oak (*Quercus incana*), occasional sand pines (*Pinus clausa*) and sand live oak (*Quercus geminata*). Because the soil had been disturbed in the early 1970s, the natural ground cover of wiregrass (*Aristida stricta*) is absent, replaced by broomsedge (*Andropogon* spp.) and several other successional species of grasses, herbs and shrubs. The same disturbance may have helped establish this dense population of *P*. *badius*, whose nests are easily spotted because the ants decorate the excavated soil disc with a layer of charcoal bits (mostly the ends of burned pine needles) [34]. The black charcoal contrasts sharply with the light-colored sand or litter.

The soil of most of Ant Heaven is classified as Ortega Sand, which formed as ridges of aeolian or sandy marine deposits on marine terraces. The soil is well-drained, with very high capacity to transmit water, and is strongly acidic throughout its profile. At the lower eastern edge of the Ant Heaven slope to the Fisher Creek wetland, the Ortega Sand grades into the very poorly drained Donovan Mucky Peat. On the west, the Ortega Sand grades into the extremely acidic Talquin Fine Sand, but little of this is included in Ant Heaven (http://websoilsurvey.nrcs.usda.gov/app/WebSoilSurvey.aspx).

### Population mapping

**Fig 1:**
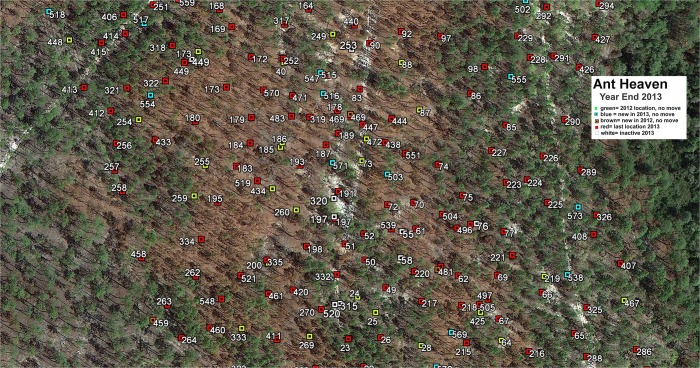
A section of Ant Heaven showing the colonies present at the end of 2013. Each colony was identified with a numbered tag, and coded for whether it did or did not move during 2013, the year it was first detected and whether it became inactive (died). Public domain aerial photograph, funded by and provided by Leon County, Florida government.

This study required the determination of the density of colonies within the site, their distribution in space, their size/depth and their frequency of relocation. Each *P*. *badius* nest at Ant Heaven was marked with a vinyl flag and a numbered metal tag, and its location recorded on a Trimble GeoExplorer CE mapping GPS instrument. Location data were differentially corrected using the base station maintained by the Department of Environmental Protection in Tallahassee, resulting in a final precision of approximately 50 cm. The population was first mapped in 2010, and the mapped area was enlarged in 2011. During surveys, areas between the easily visible flagged colonies were preferentially searched to detect new or previously undetected colonies. By the end of 2013, the tracked population numbered about 430 colonies. A section of such a survey can be seen in Fig. 1. Beginning in 2012, the population was resurveyed six times (i.e. every 4–6 weeks) between April and November of each year in order to map relocations, inactive colonies and newly detected colonies. The disc diameters of all nests were measured during each survey beginning 2012.

### Excavation and mapping of nests

Tschinkel [31, 35] reported detailed, quantitative descriptions of the architecture of *P*. *badius* nests. These data were derived from chamber by chamber excavation and 3D mapping of nests. The portion of these data essential to this study included the total chamber area, maximum nest depth and proportional distribution of chamber area by depth, and are found in S1 and S2 Tables. Chamber area was equivalent to chamber volume because chambers averaged 1 cm in height, and connecting shafts made up a small proportion of the total nest volume. Tschinkel [35] showed that the area of the disc of excavated soil was strongly predictive of the total nest volume and maximum nest depth, but did not include these in the published analysis. For this study, these disc areas were regressed against nest volume and depth to yield the regressions in Table 1 (data in S1 Table). The regressions were then used to compute nest volumes and maximum depths from disc area for each colony at each survey of Ant Heaven. Tschinkel [31] also showed that the proportion of the nest volume was similarly related to the proportion of the maximum nest depth, no matter what the size of the nest (i.e. the size-free shape of the nest was invariant). This relationship allowed computing the vertical volume occurring at six equal (50 cm) increments of depth (regressions in Table 2) for all surveyed colonies.

**Table 1:** Regressions used to estimate survey colony volumes and maximum depths.

x-variable	y-variable	intercept	slope	F	d.f.	p	R^2^
log disc area, cm^2^	Log vol., cm^3^	0.098	1.12	181	1, 17	< 0.00001	92%
Disc area, cm^2^	Max. depth	71.3	0.049	28.2	1, 17	<0.0001	60%

Data from [35].

**Table 2:** Regression equations for rate of mantling with soil from six 50 cm depth intervals.

Depth increment	Depth interval (cm)	intercept	slope
1	0–50	−301	0.717
2	50–100	−140	0.161
3	100–150	0	0.0573
4	150–200	−84.7	0.0518
5	200–250	−41.3	0.0329
6	>250	−485	0.0682

Data from [35]. The regression equations are all of the form: volume (cm^3^) = intercept + slope (total nest volume (cm^3^)). These volumes-by-depth were used in the simulation. Regressions are actually of chamber area, but because chambers average 1 cm in height, area and volume are equal.

### Nest disc spreading and dispersal

Excavated sand in the disc can be spread beyond its original extent by wind and water, especially after the ants have abandoned their nest. The rate at which this occurs was estimated by creating 20 cm, 500 g discs of undiluted, dry, fluorescent pink sand at 20 locations throughout Ant Heaven on Feb. 28, 2012. These locations were flagged and revisited occasionally for about 2 yr to determine the extent of sand dispersal. On March 22, 2012, each of the piles was photographed. On June 23, 2012, sand was collected from 10 cm^2^ areas every 10 cm from the center of each of five sand spots in 4 cardinal directions. On March 11, 2013, sand was similarly collected every 20 cm along a line running east-west through the center of five spots. The fraction of pink grains was counted under a microscope in all of these samples, and plotted against distance from the center. By the final visit on Feb. 13, 2014, two years after the placement of the discs, the sand had dispersed too much for grain counts. On this occasion, photographs under UV light showed areas where fluorescent grains could still be seen. These images did not allow meaningful quantification of sand dispersal.

### Simulation data and procedure

Simulation of biomantling required data on nest volume and nest density along with frequency, direction and distance of nest relocation. A summary of the major steps of input data preparation and sources can be found in Table 3. The data were taken from two sources: (1) Tschinkel's excavation studies [31, 35] of the subterranean nest architecture of *P*. *badius* that included the total chamber area, maximum nest depth and vertical distribution of chamber area. Tschinkel [31] collected data on disc area (S1 Table) but did not include these in his report. Here, these data allowed the disc area to be used as a proxy for nest volume and maximum depth.

**Table 3:** Summary of the major steps used to generate the input data for the simulation of biomantling.

Step Number	Procedure	Result	Source
1	Relate disc area to nest volume and depth	Regressions of disc area vs. volume, depth	Tschinkel [31, 35]
2	Determine vertical distribution of nest volume	Regression of volume of nest in six 50-cm depth increments	Tschinkel [31, 35]
3	GPS survey of Ant Heaven P. badius nests, 2012, 2013	Disc areas, nest locations, nest relocations	S3–S6 Tables
4	Application of Steps 1 and 2 to the surveys of Step 3	Estimates of nest volumes, depths and vertical volume distribution for all surveyed colonies	Steps 1–3
5	Estimation of colony life span and time to maturity	20 yr estimated lifespan (s.d. 4 yr); 6 yr growth to maturity (s.d. 2 yr).	Ant Heaven surveys, [36–37]
6	Run simulation	Estimates of the biomantling by *n* colonies in *m* areas over *t* years.	S7, S8 Tables

Details of simulation in S1 Text.

For this study, Tschinkel's data of total nest volumes [31] were classified into ten size classes in 1000 cm^3^ increments and their mean nest volume calculated. For each of these ten size categories, the sum of chamber volume within each 50-cm depth increment was calculated (S2 Table). For each of these six depth increments, volume in the increment was regressed against the mean total nest volume of the size class, yielding six regressions (Table 2) that describe the relationship of volume to absolute depth.

(2) Repeated GPS mapping of the approximately 350 colonies in the Ant Heaven population (see above) during 2012 and 2013 (six surveys each year) provided the latitude and longitude positions of all colonies and their relocations, along with measurements of their disc diameters (from which disc area was calculated) (data in S3–S5 Tables). Changes in latitude and longitude allowed the frequency, direction and distance of moves to be determined (S3–S5 Tables). At the beginning of 2013, there were 343 active colonies in the 23 ha site, giving a mean area per colony of 670 m^2^.

### Estimation of nest volume, depth and volume-distribution

For each surveyed colony, the nest volume was computed from its regression against the disc area (Table 1). These volumes were used to generate a population-wide frequency distribution. The regressions in Table 2 were then applied to these estimated nest volumes to yield the volume in each of the six depth increments for each colony in the survey. These volume-by-depth increments represented the volume of sand the ants brought to the surface from each increment during nest excavation, and were used in the simulation below.

The maximum nest depth of each surveyed colony was similarly computed from its regression against the disc area (Table 1), and used to generate a population-wide frequency distribution.

### Additional estimates needed for simulation

For a more realistic simulation, colonies should appear, grow and die on a reasonably natural schedule. A colony lifespan of 20 yr (s.d. 4 yr) was used and was based on turnover in 2 years of Ant Heaven surveys, along with published colony lifespan estimates of 17 and 40 years for *P*. *owyheei* and *P*. *occidentalis*, respectively [36–38]. Future data may revise the 20 yr lifespan estimate, but differences in mean lifespan are expected to have minor effects on the simulation outcome. Growth to colony maturity was estimated to take about 6 yr (s.d. 2 yr) based on disc size increase rates of new colonies in the Ant Heaven surveys.

### Simulation procedure

The major steps through which data from the two sources were entered into the simulation of biomantling are summarized in Table 3, and described in greater detail in the Results below and the appendix in S1 Text. The output from the simulation was visualized as a series of maps, one per generation (year) with size of the disc proportional to colony size and color showing the mix of depths from which soil in the disc was derived. These images were combined into animations showing how soil accumulates from depth to the surface, i.e. how the biomantling proceeds, coded for both quantity and depth-source of the mantling soil. The same data were also captured as an Excel file in which the volumes of soil from each of the six, 50-cm depth divisions for each nest and year were recorded.

Where appropriate, sand volumes were converted to weights by multiplying by the bulk density of dry sand, which was determined to be 1.5 kg/L.

### Data analysis

Continuous variables were analyzed by regression or ANOVA as appropriate, and transformed to stabilize the variance where necessary. Count data were analyzed by Chi-square or other non-parametric tests, except when the counts were very high in which case ANOVA was used. Because the number of colonies tracked increased during the course of this study, care was taken to use the appropriate n for computing all statistics.

## Results

**Fig 2:**
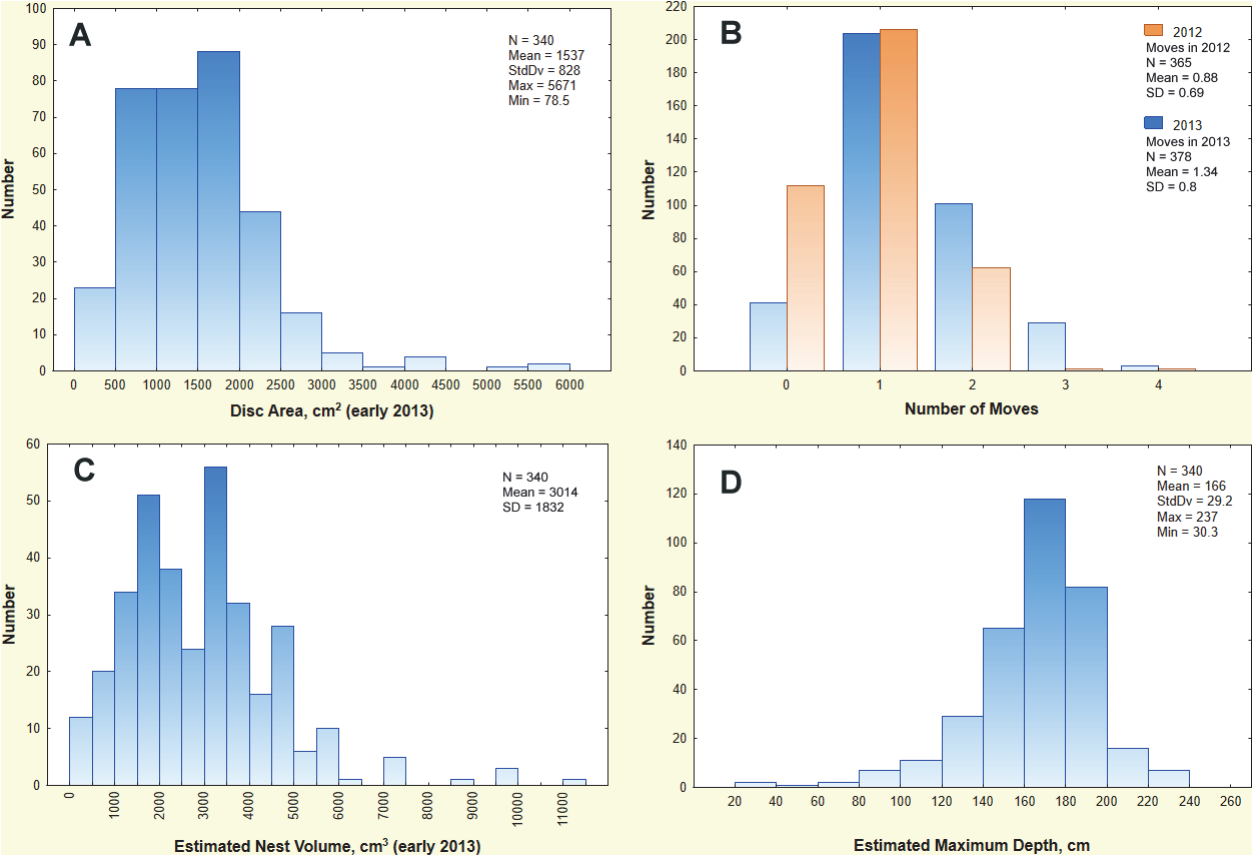
The frequency distributions of the Ant Heaven parameter values used in the simulations. A. Disc area (cm^2^) early in 2013 before colonies began to relocate. These areas therefore represent colonies that have been in place for at least 5 months over the winter and can be found in S5 Table. The distribution is best fit by a gamma distribution with a mean = 1537, S.D. = 828. B. Colonies of *P*. *badius* average about one move per year, with most moves taking place in June through October. During 2012–2013 a total of 840 moves were observed, with individual colonies moving from once per 2 years to 4 times per year. C. The total nest volume estimated from the surface nest disc ranged from about 100 cm^3^ to almost 12,000 cm^3^, with a mean of about 1500 cm^3^. D. The frequency distribution of maximum nest depth at Ant Heaven, as estimated from the relationship among nest disc area, total chamber area and maximum nest depth (Table 1).

The *P*. *badius* population at Ant Heaven averaged about 430 colonies, with "births" and "deaths" occurring every year. Fig. 1 shows a portion of a survey map for 2013. The average area occupied by an average colony at the beginning of 2013 was about 670 m^2^, and nests appeared over-dispersed (S3 Table). Frequency distribution of disc area (a proxy for nest size) was somewhat right-skewed with a range from 80 cm^2^ to about 5700 cm^2^ and a mean of 1540 cm^2^ (Fig. 2A; S6 Table). During 2012–2013, colonies moved about once a year, but moved significantly more often in 2013 than in 2012 (Fig. 2B; t_758_ = −8.36, p < 0.000001). The reason for this difference is unknown, but is treated in more detail in Tschinkel [33]. The nest volumes estimated from these disc areas (Fig. 2C, Table 1) ranged from about 100 cm^3^ to almost 12,000 cm^3^ with a mean of about 3000 cm^3^. Their frequency distribution was right skewed (skewness = 1.77, s.e. = 0.13), with a mode of about 2500 cm^3^. Very large colonies were therefore less abundant than small colonies. These nest volumes represent the volume of soil brought to the surface by the ants during nest excavation. The maximum nest depth estimated from the disc areas (Table 1) ranged from 30 cm to 245 cm, and their distribution was left-skewed (skewness = −0.45; s.e. = 0.092) with a mean of about 170 cm (s.d. = 29 cm) (Fig. 2D). Colonies deepen rapidly as they grow, but large colonies add more vertical shafts rather than deepening, thus limiting the maximum depth.

**Fig 3:**
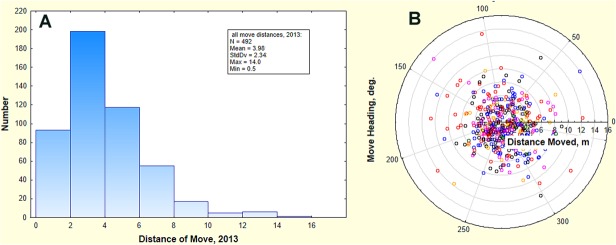
Distance and direction of moves. A. Frequency distribution of the distance moved by colonies in the Ant Heaven population. B. Colonies moved in random directions for an average of 4 m, assuring that over multiple moves, colonies performed random walks around their initial locations. Each symbol color represents a different survey during 2013.

*P*. *badius* colonies are a restless breed moving from once every 2 years to 4 times a year [33] with an average of about once a year (Fig. 2B). The average distance moved is only about 4 m, but a few colonies moved more than 10 m (Fig. 3A, [33]). Moves are in random directions (Fig. 3B), which when combined with the move distance means that over multiple moves colonies do random walks around their original locations. This zigzagging around a location justifies running simulations for relatively small areas because in spite of the frequent moves, colonies remain very local. Their gyrations bring soil to the surface from a range of depths, often from 2 m or more, even though the origin of most of this soil is relatively shallow.

**Fig 4:**
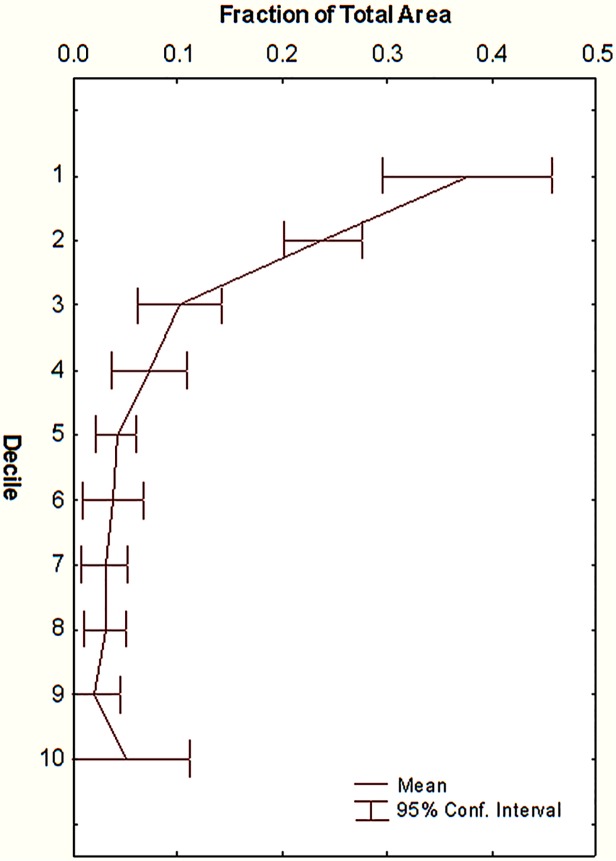
Mean size-free distribution of chamber area by decile of maximum depth. Data from Tschinkel [31].

**Fig 5:**
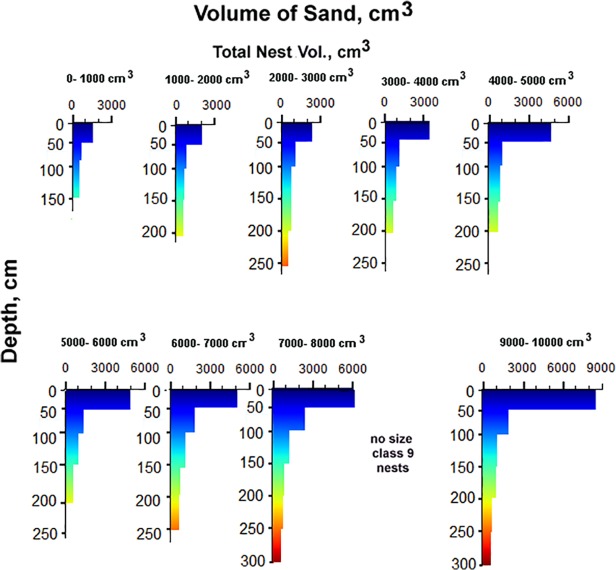
Volume of soil excavated from 50 cm depth increments. Volumes for 10 colony size classes based on 1000 cm^2^ increments of total area. Data from Tschinkel [35]. No colonies of size class 9 were found. Volumes are the sum of chamber volume within each depth increment. Because chambers average 1 cm in height, volume and area are equal. Depth from which soil was excavated is coded by color, and these colors are used to weight the depth-composition of discs in Figs. 7 and 8.

These distributions, volumes and frequencies produce the overall rates of biomantling. What remains to be estimated is the relative amount of soil brought up from various depths. To this end, Tschinkel [31] showed that the "size-free shape" of the nest does not change with nest size (i.e. the proportion of total nest area (volume) found at each proportion of maximum depth is invariant) (Fig. 4). Therefore, given a total nest volume derived from the disc area, the distribution of this volume in relation to depth can be estimated from the relationship in Fig. 4. For the simulations, the amount of soil brought to the surface from each of six 50 cm depth regions was estimated for ten nest size classes (data from [35] in S2 Table) ranging from less than 1000 cm^3^ to about 10,000 cm^3^ and depths to almost 3 m. These are shown in Fig. 5, and were used to generate the equations in Table 2. The depths are coded by color, and these colors are used for depth-weighted composition of disc soil in the simulations below.

**Fig 6:**
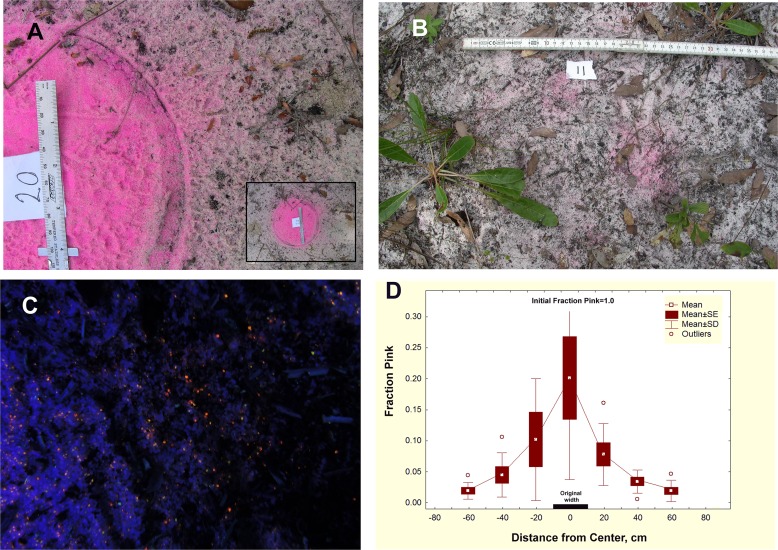
The spreading experiment after three elapsed times. A. Representative disc of colored sand on March 20, 2012, 20 days after emplacement. Note the haze of pink grains that has dispersed well beyond the original disc boundary. The agents of transport are probably wind and water, but possibly also animals. Note the raindrop pitting especially visible on the disc. Inset shows entire disc. B. Appearance Sept. 21, 2012. approx. 7 months after emplacement. C. A disc of fluorescent sand 2 years after emplacement, under UV light in situ. The color of the disc was no longer visible without the aid of UV fluorescence. D. Distribution of pink grains in the spreading experiment, March 11, 2013, one year after emplacement of 500 g of 100% pink sand in a disc 20 cm in diameter. Within a year, significant amounts of pink sand have spread more than 50 cm from the edge of the original disc.

Excavated soil piled into discs is not stable, as was shown in the spreading experiment with fluorescent pink sand. The dispersal of 20 cm diameter discs of pink sand (n = 20) was followed over two years. Obvious spreading had occurred within 20 days (Fig. 6A), and by 7 months, most discs were recognizable as mere pink blushes (Fig. 6B). By the end of one year, pink sand had spread more than 50 cm from the edge of the original disc (Fig. 6D), and by two years, pink grains were detectable only through the use of a UV light (Fig. 6C). Fig. 6D shows that both pink and native sand migrated, diluting the original disc from 100% pink to only about 20%. The mobile layer in this habitat (in the absence of disturbance) is generally 2–5 mm thick, underlain by a layer in which grains are bound by (probably) fungus, algae and plant roots into a stable, grey layer. Pink grains also became incorporated into this more stable layer.

The effect of this surface migration is to form the excavated disc soil into a uniformly spread layer whose thickness can be calculated from the total volume excavated and the area per colony. In the Ant Heaven population, the average colony occupied an area of 670 m^2^. For simulation purposes an area 25 m x 25 m (625 m^2^) was used, as density was higher than average in some areas.

### Simulations

Using the data described above, simulations proceeded as follows: (1) an initial simulation area was chosen (e.g. 25 m x 25 m). An initial "colony" location within the area based on x and y coordinates was randomly selected within the simulation area; (2) all "colonies" lived 20 years (s.d. = 4 yr) at which time they were replaced at a randomly-selected x, y position by new, small colonies of age 0, disc radius of 7.5 cm and disc area = pi * r^2^; (3) a growth period drawn randomly from a normal distribution with a mean of 6 yr (s.d. = 2 yr); (4) a final mature size calculated as 100 * a random number from a log normal distribution with mean 3.3 and s.d. = 0.6; These sizes are based on survey data (Fig. 2), and can be found in S3–S4, S6 Tables; (5) A stable average population density through time; (6) These "colonies" excavated nests of a volume and maximum depth determined from the regression of real volumes vs. disc areas (Table 1; Fig. 2C-D). (7) The depth of the excavated "soil" brought to the surface from each 50-cm depth increment was determined from the regressions in Table 2 based on the patterns in Fig. 5. (8) colonies were "moved" once a year in a random direction (Fig. 3B) taken from a random number between 0 and 2pi (in radians), and new x, y coordinates drawn randomly from a normal distribution with mean 3.9 m and s.d. = 3.15 m (Fig. 3A). If the move distance placed them outside the simulation area, a new one immigrated in. The simulation was run for a specified number of "generations" (= years). More details of the simulation can be found in the appendix in S1 Text.

**Fig 7:**
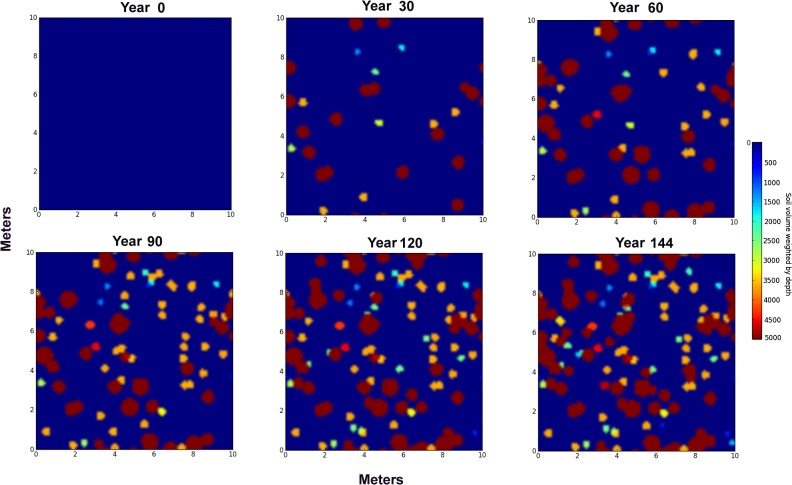
Simulation of a single colony in 100 m^2^. Simulation was run until 25% of the area was covered by discs, about 145 years. Densities this high probably rarely occur in nature, but illustrate the principles of the simulation. Blue indicates a shallow source of soil, while red indicates admixture of deepest layers. Colonies move a mean of 4 m per year in random directions (see text for details).

The simulation program output included a set of images showing the location, size and depth-source of the nest discs for every year, and accumulated these data in an Excel file for further analysis. The images were also combined into animations showing the year by year progress of the mantling of the surface with nest discs. The principles of this simulation are made obvious in a run with one colony occupying 100 m^2^ (an unnaturally high density) (Fig. 7). The effects of the annual moves, the lifespan, growth and maximum depth of the colony can readily be seen in the increase of disc size as the "colony" grows, change in disc color as maximum nest depth increases and deeper soils are part of the disc-mix. Upon colony "death", it is replaced by a "colony" with a smaller disc composed of shallower soils.

**Fig 8:**
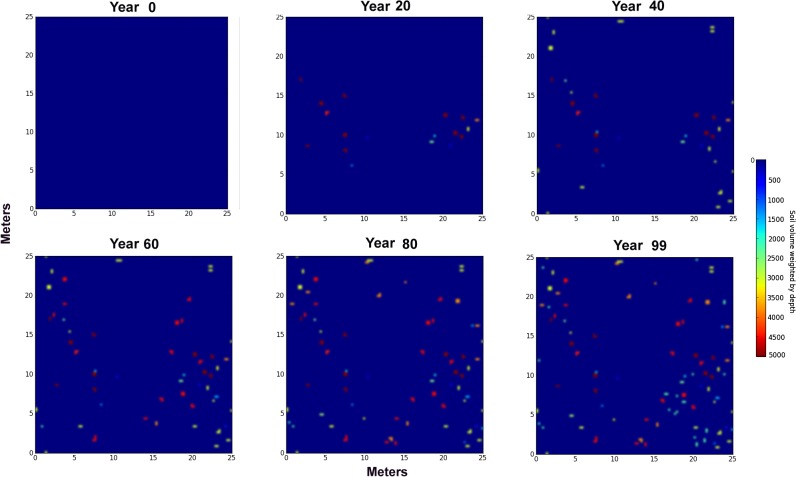
A representative simulation of disc locations of one colony in 625 m^2^. Increments of 20 years shown, with colors showing depth-weighted source of soil. This is the approximate natural density of colonies. Blue indicates a shallow source of soil, while red indicates admixture of deepest layers. Colonies move a mean of 4 m per year in random directions (see text for details).

**Fig 9:**
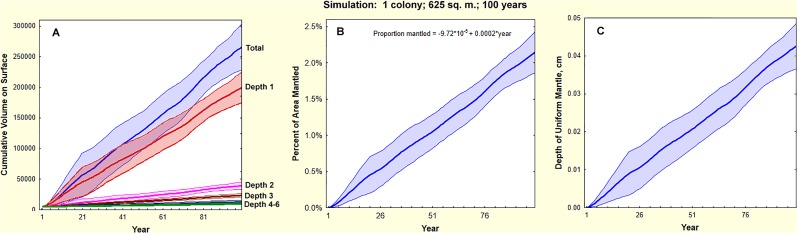
Simulation of mantling by one colony in an average territory over 100 years. A. The cumulative volume (cm^3^) of soil brought to the surface from increasing depth (only 5 of 6 depth categories are shown) and the total over one century. By far the most soil was brought up from the shallowest depth where the largest chambers are located. Regression equations for each depth category are given in Table 2, data in S7, S8 Tables. Each colony mantles the surface with about 200 L per century from depth 1, 35 L from depth 2, then decreasing to 5 L from depth 5. B. the cumulative proportion of the area covered by nest discs. By the end of a century, nest discs would have covered over 2% of the area, but wind, rain and animals would have spread these out much more uniformly. C. The depth of mantled soil if it were spread uniformly over the area. Approximately 0.35 to 0.5 cm accumulate per millennium, with a mean of about 0.43 cm.

Fig. 8 shows panels from a simulation of a single colony in 625 m^2^ for 100 years. A sample animation can be viewed in the S1 Animation. Twenty runs of this simulation were used to compute mean values for several measures, including the cumulative volume of soil brought to the surface (Fig. 9A). The source of this soil is predominately from the shallower depths (Depth 1, 0–50 cm), with decreasing proportions from greater depths. Fig. 9B shows the percent of the area covered by nest discs and Fig. 9C the thickness of the mantled soil layer if it were evenly spread over the area. At the end of a millennium, the colony (and its replacements) will have brought up about 2660 L (s.d. = 370 L) covering about 21% (s.d. = 2.9%) of the area, which, when spread evenly would make a layer 0.43 cm (s.d. = 0.05 cm) thick.

**Fig 10:**
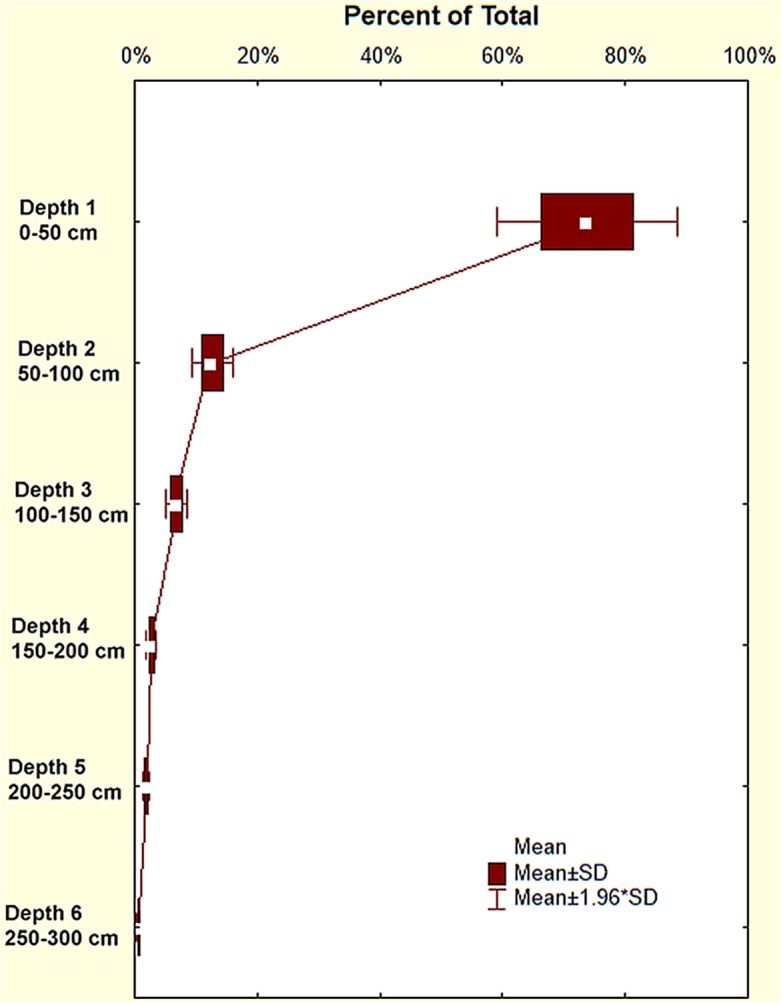
Simulation of the proportion of the mantled soil originating from each depth. These values change little over the 100 years of the simulation because the "shape" of the nests was assumed to be the average, and was thus invariant.

Of the 2660 L brought to the surface by each colony in a millennium (Fig. 9A), 1980 L (s.d. = 250) came from the shallowest depth increment, but only 34 L (s.d. = 18 L) and 14 L came from depths 5 (200–250 cm) and 6 (>250 cm), respectively. Because the size-free shape of the nests is invariant, the mean proportion of soil from the depth increments is independent of time and is shown in Fig. 10.

Projecting these Ant Heaven results to a per ha rate, an average of 16 colonies per ha together deposit 42,600 L of sand on the surface. At a bulk density of packed, dry sand at 1.5 kg/L, this is 63,900 kg or 63.9 metric tons per ha. Of this, about 1.2 tons (approximately 2%) came from depths greater than 200 cm.

## Discussion

Over a millennium and multiple generations, the Ant Heaven population of Florida harvester ant, *Pogonomyrmex badius* will move about 6.4 kg of soil (4.3 L) per square meter, or about 64 metric tons per ha, from depths to the soil surface. Spread evenly, this amounts to a layer 0.43 cm thick. *P*. *badius* is thus a significant agent of biomantling and bioturbation in the sandhills habitats in which it occurs, affecting the cycling of soil nutrients in this nutrient-poor habitat. Much of this mantling is the result of frequent nest relocation, which averages about once a year [33]. During these moves, each colony of *P*. *badius* excavates a new nest as large or larger than the old one in 4–6 days, depositing an average of 4.8 kg and up to 20 kg of sand on the surface.

The excavation and biomantling activity of *P*. *badius* must be seen in the context of the rich and dense fauna of ground-nesting ants [39] and other animals that burrow in the soil of this site. Taken together, ants are probably the most important biomantling and bioturbation agents in these coastal plains habitats, probably exceeding vertebrates such as rodents and gopher tortoises [40]. Although *P*. *badius* nests are unusually large, their density is relatively low. A few species, such as *Prenolepis imparis*, make nests far deeper than *P*. *badius*, while the shallower, smaller nests (∼0.5–1.5 m) of others, including *Trachymyrmex septentrionalis*, *Nylanderia arenivaga*, *Pheidole dentata*, *Pheidole morrisi*, *Camponotus socius* [41, 42] are more abundant than those of *P*. *badius*. The fungus gardener, *T*. *septentrionalis* is particularly common, occurring at densities of 1000 nests per ha or more. Each spring, these ants bring 0.5 to 1.5 metric tons of sand per ha to the surface, mantling the soil surface with over 6 cm of soil per millennium [28]. Remarkably, this rate is similar to that of the tropical leafcutter ants of the genus *Atta* with their colossal (but rare) nests, illustrating that abundance can more than make up for small size. The mantling rate of *T*. *septentrionalis* also rivals estimates for all ants together in some ecosystems [43], and is almost 150 times the mantling rate of *P*. *badius*. Although each individual colony of *P*. *badius* brings far more soil (from 0.1 to 12 L) to the surface each year than a *T*. *septentrionalis* colony, their low density means that their total contribution to biomantling is relatively low. On the other hand, in contrast to many of these more abundant ant species, a substantial fraction (10–15%) of the soil mantle produced by *P*. *badius* originates from deeper layers (>1 m). Most ant species build nests that have the largest proportion of the total volume near the surface (are top-heavy) [31, 41], so that most bioturbation involves mostly the near-surface soil.

Historically in the north temperate zone, earthworms have been considered primary agents of bioturbation (reviewed in [3, 22]. In England [1] and Sweden [44], earthworm bioturbation far exceeds that of ants, making up 80 to 100% of total bioturbation. In contrast, earthworms are a minor component of bioturbation in the southeastern USA, especially in the coastal plains longleaf pine forest. Here, it seems likely that ants are the major bioturbators, especially in light of the fact that a single species within this dense ant community, *Trachymyrmex septentrionalis*, deposits 0.5 to 1.5 tons of soil per ha on the surface every year. The contributions of the other abundant ant species in this community (with the exception of *P*. *badius*) are currently unknown, but probably substantial, bringing the total subsurface soil brought to the surface even higher.

Surface deposition does not tell the whole story of bioturbation. Laboratory experiments by Halfen and Hasiotis [20, 21] showed that about half of the soil excavated by *P*. *occidentalis* was deposited below the surface. Whether this is representative of natural colonies is uncertain, but Humphreys and Field [45] included subsurface mixing of soils, and Rink et al. [18] showed that subsurface deposition of deeper soil by *P*. *badius* occurs most frequently in the upper 30 cm of the nest, but such deposition is only a small fraction of the total deposited on the surface. *P*. *badius* also uses deeper soil to backfill chambers, as does *P*. *occidentalis* [21]. Less expected is that these ants also move soil downward, especially from levels below about 60 cm [18]. All of these movements and deposition—upward, downward and surface—make up the total bioturbation, although precise quantification of the upward and downward components are presently lacking.

Soil deposits on the surface are inherently mobile and are spread laterally by rainwash, wind [8] and animals, with rates of movement increasing down slope [7, 46]. Loose colored sand placed on the surface of our sandhills site began its lateral spread immediately, so that within a year, most of these colored sand patches were barely (or not) discernible, but colored grains were detectable up to 1 m from their original location. Taken over centuries or millennia, this lateral mixing would spread ant-mounded sand evenly over the site. In more complex soils than the almost pure, horizon-less sand of the Florida coastal plain, lateral spread probably involves sorting, with some components spreading more rapidly than others [12]. The ghosts of *P*. *badius* nests, though visible for up to a year or more, eventually become level and indistinguishable from the surrounding sand. In our sandhill site in undisturbed spots, a mobile layer a few mm thick covered a less mobile grey layer bound by (perhaps) fungus and algae. Repeated traffic converted such areas into loose layers of sand several cm deep, and in the extreme after long rainless periods, into sand traps capable of impeding car traffic.

Literature mantling rates for various species of ants or whole ant communities vary enormously, from low values in the vicinity of 0.1 tons/ha/yr to high values of 10 tons/ha/yr (Table 4). The reasons for this variation are probably several, with the size and density of nests probably being the most important, and methods of estimation possibly contributing. Many estimates were extrapolated from mounding rates, that is, the weight or volume of soil piled up by the observed nests, sometimes in very limited areas (e.g. [3]). Authors do not often report estimates of nest densities over large areas of habitat. Ants are often highly sensitive to small variations in habitat characteristics, leading to large spatial variation in nest density. For example, Tschinkel et al. [47] found that ant species segregated along a very subtle elevation and depth-to-groundwater gradient in the Florida flatwoods. Such heterogeneity is undoubtedly common, and could cause large errors in bioturbation estimates if these assumed uniform nest density.

**Table 4:** Literature estimates of mounding rates and bioturbation by ants.

Species	Mounding rate (kg/m^2^/yr)	Layer thickness/time	Was nest density estimated?	Reference
*Atta texana*		0.6–2+m	no	[3]
*Lasius neoniger Tetramorium caespitum*	0.170	90 cm /1000 yr	no	[3]
*Aphaenogaster sp*.	0.22–0.47		Yes	[6]
*Aphaenogaster barbigula*	0.34	0.28 mm/yr	In quadrats	[11]
*Aph*. *longiceps*	0.84		In quadrats	[22]
*Aph*. *longiceps*	0.028		unknown	[22]*
*Aph*. *longiceps*	0.52–0.58		unknown	[22]*
*Aph*. *longiceps*	0.27–.018		Yes	[5]
*Myrmica spp*.	0.013–0.075		Yes	[44]
*Trachymyrmex septentrionalis*	0.5–1.0	6.3 cm /1000 yr	yes	[28]
*Formica* sp.	0.83–1.13	2 cm/10 yr	unknown	[22]*
*Formica cinerea*	4.9 (from 0.02 m^3^ /mound)		1531/ha	[29]
*P badius*	0.011		yes	This paper
Ants	0.036–0.066		unknown	[5]
Ant community	0.035–0.042		Yes	[27]
Ant community	0.01–0.037		yes, 0.5 to >2% coverage	[6]
Ants	0.0021–0.0086			[10]
World ants	0.1–0.5			[22]
termites	0.075–0.41			[10]
All ants	0.042–1.0			[43]
burial of tiles		250–400 mm/1000 yr	n/a	[17]

Authors did not always report the density of nests over larger areas, thus some extrapolations may be inaccurate. Rates are reported here as kg/m^2^/yr. Data in tons/ha/yr have been converted to kg/m^2^/yr. Some data (see bottom of table) were taken from review rather than the original source.

* Data from Mitchell 1988; Humphreys and Mitchell 1983; Salem and Hole 1968; all as reported in [22].

The present study is exceptional in that the nest density and size distribution for the entire 23 ha site were determined with a high degree of confidence. In comparison with other studies of ant bioturbation, this one is based on the actual architecture of the subterranean nests, their spatial density and the frequency of their relocation, rather than simply the rate of mounding of soil on the surface. This study thus gives a better account of the source of the mounded soil, as well as providing biomantling estimates that are based on realistic biological knowledge.

## Supporting Information

S1 Table Basic architectural features of colonies. The data are taken from [31].
(XLS)

S2 Table Nest volumes in 50 cm depth increments. Data used for these computation are from [31].
(XLS)

S3 Table Location and size data for all colonies present in 2012 at Ant Heaven. Latitude and longitude of each colony at each survey, along with disc size of old and live nest.
(XLS)

S4 Table Location and size data for all colonies present in 2013 at Ant Heaven. Latitude and longitude of each colony at each survey, along with disc size of old and live nest, direction and distance of move.
(XLS)

S5 Table Ant Heaven survey data for 2012–2013. Data as recorded on the GPS.
(XLS)

S6 Table Disc area of all Ant Heaven nests at the beginning of 2013. (XLS)

S7 Table Simulation output for biomantling and area of 625 m2 or 100 years. (XLS)

S8 Table Continuation of S6 Table. (XLS)

S1 Animation Video animation of the output of a simulation. The video shows the year-by-year deposition of soil on the surface, with deposited soil color-coded for source depth.
(MPG)

S1 Text Details of the simulation steps and inputs. (DOCX)
