# Supplementary material for: Biomantling and Bioturbation by Colonies of the Florida Harvester Ant, Pogonomyrmex badius
Source: PLoS One. 2015 Mar 20;10(3):e0120407. doi: 10.1371/journal.pone.0120407 (PMC4368782; doi:10.1371/journal.pone.0120407)
Supplement: S1 Text — (DOCX) [file pone.0120407.s010.docx]

**Appendix 1. Stepwise description of simulation**

Once initiated, the program creates abstractions called 'nests' which have certain attributes:

1. x and y coordinates (initially random numbers taken from the range specified by the user)

2. an age (starting at 0)

3. whether the nest is 'extinct' (starts at false)

4. a centimeter radius (starts at 7.5)

5. a surface area (radius^2 * pi)

6. a total chamber area (log chamber area = -0.098 + 1.12 log disc area)

7. a lifespan (a random number from a normal distribution with mean 20 and stdv 4)

8. a growth period (a random number from a normal distribution with mean 6 and stdv 2)

9. a final 'adult' size (100 * a random number from a log normal distribution with mean 3.3 and stdv 0.6)

The nest has volumes associated with each of the six depth categories as derived from the total chamber area via:

V _depth1_ = -301 + 0.717 * (chamber area)

V _depth2_ = -140 + 0.161 * (chamber area)

V _depth3_ = 0 + 0.0573 * (chamber area)

V _depth4_ = -84.71 + 0.0519 * (chamber area)

V _depth5_ = -41.3 + 0.0329 * (chamber area)

V _depth6_ = -485 + 0.0682 * (chamber area)

If these equations generate negative values they are replaced with 0. These numbers will not add up exactly to the chamber area, so they are summed into a total area, the proportion of the total taken by each one is found, and then this proportion is applied to the chamber area.

e.g. adjusted depth1 = (depth1 / total area) * chamber area

Then each nest is given a final weighted depth from these adjusted depths:

weighted depth = depth1 + 2*depth2 + 3*depth3 + 4*depth4 + 5*depth5 + 6*depth6

The program then prints an image matrix that shows the position, radius and weighted depth of each nest. Positions within the coordinate plane where there is no nest (i.e. outside of the radii extending from the coordinates of each nest) are set as 0. Positions within the radius of a nest coordinate are assigned the weighted depth value. This matrix is then displayed on the screen using the common method 'imshow'.

After the drawing, the program iterates through a new generation and each nest is 'migrated.' The migration involves displacing the x and y coordinates by a magnitude taken from a normal distribution with mean 3.9 and stdv 3.15. (If the magnitude is below 0 a new number is drawn.) The direction is taken from a random number between 0 and 2*pi. Periodic boundary conditions are assumed (i.e. if a nest emigrates from the study area, a new one immigrates in).

Then the nest's life history traits are updated. The age is increased by 1. If the nest is younger than its growth period, the chamber area is increased by taking the final adult size and dividing by the growth period. (It is assumed each nest undergoes linear growth through this period.) If the nest is older, its chamber area does not change. The radius is derived back from the chamber area. If the nest's age has increased past its lifespan, the 'extinct' attribute changes from 'false' to 'true' and the nest is replaced by a new nest with all new attributes. (Note that there is an assumption of no correlation between a nest's final size and the length of its growth period.)

After each generation the matrix is updated and redrawn.

The matrix is also checked for the percentage of elements which are non-zero. This is interpreted as the percentage of the total area that has been disturbed. This information, along with the total chamber area (summed across all nests) and the subtotals for each depth category are reported in the Excel spreadsheet with each generation as a new row.

OUTPUT:

The program will create a new folder in the directory it was run from. This will be date- and time- stamped so that each simulation is stored in a distinct directory. The folder will contain an .mpg video file of the animation as well as an Excel workbook tabulating the volumes of soil per depth layer with each generation as well as the proportion of the range that has been disturbed with each generation. Another nested directory will contain all of the image files used for the animation in case that is of interest. Temporary files will be created in the simulation directory, which will be deleted after the animation is made. If for some reason the simulation is interrupted, the temporary files will not be deleted. If you see many files labelled "_tmp###.png" and the program is not being used, these may be deleted.
